# The prevalence of anxiety symptoms/disorders in cancer patients: a meta-analysis

**DOI:** 10.3389/fpsyt.2024.1422540

**Published:** 2024-11-15

**Authors:** Sohrab Amiri

**Affiliations:** Spiritual Health Research Center, Life Style Institute, Baqiyatallah University of Medical Sciences, Tehran, Iran

**Keywords:** anxiety, cancer patients, meta-analysis, systematic review, anxiety symptoms

## Abstract

**Objective:**

Cancer can have negative effects on mental health. The aim of this study was to investigate the prevalence of anxiety symptoms/disorders in cancer patients’ worldwide using meta-analysis.

**Methods:**

The study population was cancer patients who had cancer at the time of the study. The outcome studied in this study was anxiety symptoms/disorders. PubMed and Scopus were searched based on the syntax of keywords, this search was limited to articles published in English until September 2021. For this meta-analysis, data on the prevalence of anxiety were first extracted for each of the eligible studies. The random-effects method was used for the pool of all studies. Subgroup analysis was performed based on sex, anxiety disorders, cancer site, and continents. Heterogeneity in the studies was also assessed.

**Result:**

After evaluating and screening the studies, eighty-four studies were included in the meta-analysis. Prevalence of anxiety symptoms/disorders in cancer patients showed that this prevalence is 23% (*I^2^ =* 99.59) in the 95% confidence interval between 22-25%. This prevalence was 20% (*I* ^2^ = 96.06%) in the 95% confidence interval between 15-24% in men and this prevalence is 31% (*I* ^2^ = 99.72%) in the 95% confidence interval between 28-34% in women. The highest prevalence of anxiety was in patients with ovarian, breast, and lung cancers.

**Discussion:**

It showed a high prevalence of anxiety symptoms/disorders in cancer patients, in addition to therapeutic interventions for cancer, the necessary interventions should be made on the anxiety of these patients. Methodological limitation was the heterogeneity between the studies included in the meta-analysis. Some types of cancer sites could not be studied because the number of studies was small or the site of cancer was not identified.

## Introduction

Mental disorders are among the leading causes of disease burden in the world so that a global study in 2019 showed that one of the two debilitating mental disorders was anxiety disorders, which was classified among the top 25 disease burden factors in 2019 (1, 2). Anxiety disorders continue to be one of the most common mental disorders in the world (3). Accordingly, a study by the Global Burden of Disease shows that anxiety disorders are responsible for 26.68 million disability-adjusted life years (DALYs) (4). Studies show that anxiety disorders are more common than other mental disorders such as mood disorders, substance abuse, and impulse control disorders (5, 6). An examination of the prevalence of anxiety disorders shows that the prevalence of these disorders varies in different countries (7). According to the report published by the World Health Organization, anxiety symptoms often begin in childhood and adolescence (8). Currently, studies have reported a prevalence of anxiety disorders of 7.3%, ranging from 4.8% to 10.9% (9, 10).

There are gender differences in the prevalence of anxiety so that women are more likely to be affected by anxiety compared to men (11). Lifetime Generalized anxiety disorder prevalence was 3.7%, 12-month prevalence was 1.8%, and 1-month prevalence was 0.8% (3). The prevalence of generalized anxiety disorder was higher in high income countries (5%) than low income countries (1.6%) (3). Some of factors associated with the risk of developing anxiety disorders, including high body mass index (12–14), diabetic (15–17), stroke (18, 19), and personality and social risk factors (20). Cancer is diseases associated with anxiety (21).

Cancer is one of the leading causes of death in the world (22). A global study published in 2016 shows that cancer is responsible for 213 million DALYs and 8.9 million deaths (23, 24). Between 2006 and 2016, cancer cases increased by 28%, the lowest increase was in countries with high sociodemographic index (23). In general, lung cancer is the most common type of cancer between the sexes (25). The most common type of cancer among men is prostate cancer, while the most common type of cancer among women is breast cancer (23). It is estimated that the number of cancers will increase to 28.4 million by 2040, which shows a 47% increase compared to 2020 (26). A range of factors has been suggested to increase the risk of cancer, including diet (27–29), smoking and alcohol use (30–32), aging (33, 34), psychological factors (35–37). Considering the effect of cancer on the dimensions of mental health (38, 39), studies have investigated the prevalence of mental disorders in cancer patients (40–42).

The 12-month prevalence of mental disorders in cancer patients was reported to be 39.4%, the most common of which were anxiety disorders (15.8%), followed by mood disorders (12.5%) and somatoform disorders (9.5%) (43). Extensive systematic review and meta-analysis studies have examined the prevalence of mental disorders, especially anxiety disorders, in cancer patients (44–50). Although studies examining the prevalence of anxiety disorders in cancer patients have become widespread, and systematic review and meta-analysis studies have been conducted in this area, several points required a new global study. First, fewer studies have been conducted on the prevalence of anxiety disorders, and most studies have studied a combination of mental disorders, and less distinction has been made between types of mental disorders. Second, in previous studies, each study often dealt with some types of cancer, and therefore different cancer sites have been less studied, and therefore a comprehensive view of different types of cancer is needed. Third, it is necessary to differentiate between men and women based on the types of cancer and anxiety disorders because types of cancer and anxiety disorders have different prevalence in men and women. Fourth, in studying the prevalence of anxiety disorders in cancer patients, a distinction should be made between cancer patients and cancer survivors. In this study, the focus was on cancer patients and not cancer survivors.

Based on what was stated, the aim of the present study was to investigate the global prevalence of anxiety symptoms/disorders in cancer patients and also to investigate the prevalence of anxiety based on sex, cancer site, type of anxiety disorder, and continents by conducting a meta-analysis.

## Method

### Protocol

The Preferred Reporting Items for Systematic reviews and Meta-Analyses (PRISMA) (51) guide was used for this study.

### Information sources

Two databases, including PubMed and Scopus, were searched. This search was limited to articles published in English until September 2021. This search examined articles that were available online.

### Search strategy

This search based on the syntax of keywords in **Appendix 1**; For example: The search strategy included terms such as ‘cancer’, ‘anxiety disorders’, ‘anxiety symptoms’, combined with Boolean operators.

### Selection criteria

In the present study, a set of inclusion and exclusion criteria was considered. The study population was cancer patients who had cancer at the time of the study. The outcome studied in this study was anxiety disorders or anxiety symptoms (Anxiety measurement included anxiety scales or anxiety diagnostic interviews). For the present study, cross-sectional, prospective and longitudinal studies (at baseline) were selected as the eligible study design. The following studies were not eligible: 1) Studies that have studied incidence rate. 2) Studies that have studied anxiety and depression together. 3) Studies on cancer survivors. 4) Studies with a sample size of fewer than 100 participants. 5) Review studies, studies with insufficient information to calculate the prevalence, and studies with the same database.

### Data extraction

The extracted information included the characteristics of the authors, the year of the study, the demographic characteristics of the study population, as well as the methodological characteristics and results of each study. One researcher was responsible to data extraction.

### Qualitative assessment

For quality evaluation, EPHPP tool (52, 53) was used, which in this study, three adjusted dimensions were used. These dimensions included selection bias, data collection method bias, and withdrawals/dropouts, and missing bias.

### Meta-analysis

For this meta-analysis, data on the prevalence of anxiety symptoms/disorders were first extracted for each of the eligible studies. After extracting the data for each study, some of the studies had several effect sizes, in which the average effect size was calculated. In some studies that used several anxiety measurement tools, one tool was selected. The random effects method was used for the pool of all studies in the form of meta-analysis. Subgroup analysis was performed based on sex, anxiety disorders, cancer site, and continents. Also, in the end, the degree of heterogeneity in the studies was examined using *I* ^2^ and χ^2^ (54, 55). Data analysis in this study was using Stata-14 (Stata Corp., College Station, TX).

## Results

### Selected studies

**Figure 1:**
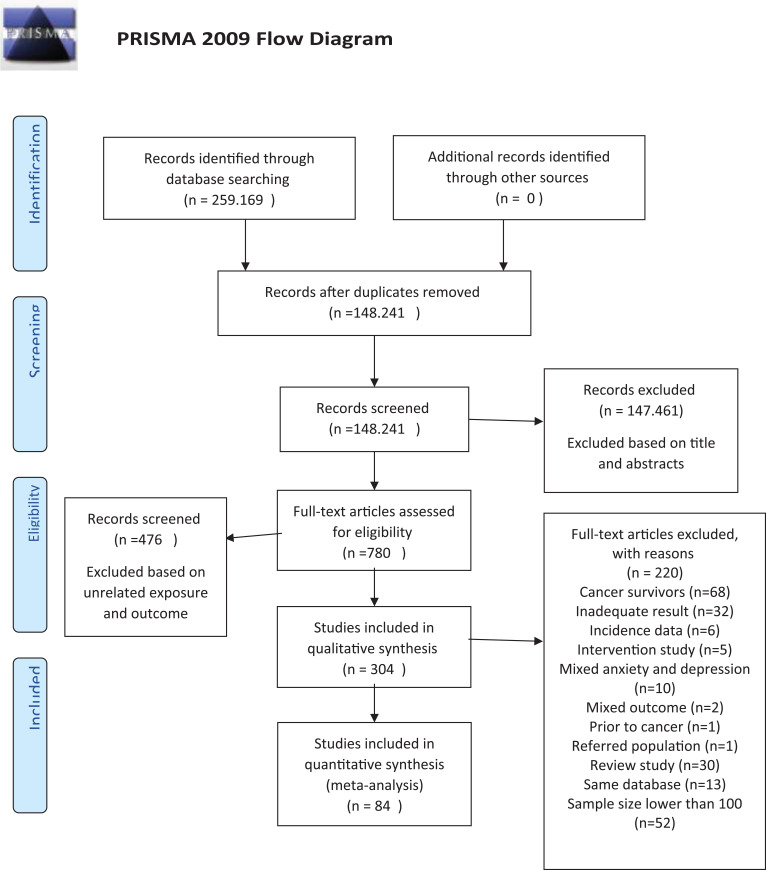
Flowchart of included studies. From: Moher et al. (150). For more information, visit www.prisma-statement.org.

**Figure 1** shows the selection and screening steps for articles. After screening, 84 eligible articles (43, 56–139) were included in the current meta-analysis (**Table 1**).

**Table 1:** Studies included in the meta-analysis.

First author and year of publication	Country	Study design	Age	Sex	Sample size in anzlysis	Cancer Cite	Anxiety symptoms/disorders	Anxiety measure	Quality assessment: Risk of bias			Results Sample (Event)
									Selection	Data collection method	Withdrawals/ dropouts and missing	
Abuelgasim 2016 (56)	Saudi Arabia	Cross-sectional	27-64	42.7% women	211	Hematological cancers	Generalized Anxiety Disorder	Generalized Anxiety Disorder-7	Moderate	Low	Low	Total 211 (47) Men 121 (23) Women 90 (24)
Ahmed 2018 (57)	Saudi Arabia	Cross-sectional	≥18	57.6% women	375	breast cancer, colon cancer, lung cancer, other types of cancer	Anxiety >3	Depression Anxiety Stress Scale	Moderate	Low	Low	Total 375 (197) Men 154 (66) Women 216 (127)
Akyol 2015 (58)	Turkey	Cross-sectional	28-76	31% women	105	Colorectal cancer	Anxiety Score>= 8	HADS	High	Low	Low	105 (30)
Anuk 2019 (59)	Turkey	Retrospective	16-87	62% women	566	breast cancer, lung cancer, gastrointestinal cancers, head and neck cancer, gynecologic cancers	Anxiety	DSM-IV-TR	Low	Low	Low	Total 566 (55) Anxiety disorder 566 (27) Common anxiety disorder 566 (13) Panic disorder 566 (3) The claustrophobia 566 (4) Obsessive-compulsive disorder 566 (1) Somatoform Disorder 566 (7) Men 215 (15) Women 351 (40)
Bergerot 2017 (60)	Brazil	Prospective	54.4 ±15.5	69.9% women	548	Breast, Gastrointestinal, Genitourinary, Gynecological, Hematological, lung and other	Anxiety Score>= 8	HADS	Low	Low	Low	548 (203)
Blázquez 2016 (61)	Spain	Longitudinal	58.21 ±13.3	55.3% women	103	Breast, Neck and head, Genital system, Digestive system, Respiratory system and other	Anxiety	Mini-International Neuropsychiatric Interview HADS	High	Low	Low	Total 103 (17) Panic 103 (10) Agoraphobia 103 (7) Generalized anxiety 103 (3)
Borstelmann 2015 (62)	USA	Prospective cohort	17-40	Women	675	breast cancer	Anxiety Score>=11	HADS	Low	Low	Low	675 (158)
Cardoso 2016 (63)	Portugal	Cross-sectional	18-83	50.7% women	270	Colorectal, lung, breast, uterus/ovary	Anxiety Score>= 8	HADS	Moderate	Low	Low	Total 270 (81) Men 133 (26) Women 137 (55)
Chambers 2015 (64)	Australia	Cross-sectional	≥18	48.3% women	151	lung cancer	Anxiety Score>= 8	HADS	Moderate	Low	Low	Total 151 (74) Men 78 (33) Women 73 (41)
Champagne 2016 (65)	France	Longitudinal	56.4 ±10.8	Women	120	breast cancer	GAD	Semi-structured interview	Moderate	Low	Low	120 (17)
Civilotti 2021 (66)	Italy	Cross-sectional	≥18	Women	478	breast cancer	Anxiety Score>= 8	HADS	Low	Low	Low	478 (189)
Costa-Requena 2010 (67)	Spain	Cross-sectional	18-80	52% women	494	Breast, Gastrointestinal, Respiratory, Head and neck, Other solid tumours, Not solid tumours	PTSD	PTSD Checklist-Civilian version	Low	Low	Low	494 (49)
Coyne 2004 (68)	USA	Unknown	34-89	Women	113	breast cancer	Anxiety	Structured clinical interview	Moderate	Low	Moderate	113 (7)
Daştan 2011 (69)	Turkey	Cross-sectional	≥20	Women	123	Breast Cancer	Anxiety Score>= 8	HADS	High	Low	Moderate	123 (43)**
Dinkel 2014 (70)	Germany	Cross-sectional	58.4± 12.8	35.2% women	341	Gastrointestinal Hematological	Anxiety	Structured Clinical Interview	Low	Low	Moderate	Total 341 (60) Generalized anxiety disorder 341 (7) Panic disorder with agoraphobia 341 (9) Panic disorder without agoraphobia 341 (7) Agoraphobia without history of panic disorder 341 (4) Social phobia 341 (12) Specific phobia 341 (16) Obsessive–compulsive disorder 341 (6) Posttraumatic stress disorder 341 (12)
Ene 2006 (71)	Sweden	Longitudinal	63.1 ± 5.2	Men	123	prostate cancer	Anxiety	HADS	Moderate	Low	High	123 (28)**
Geue 2018 (72)	Germany	Cross-sectional	≥15	64.6% women	302	breast cancer, hematologic neoplasm, colon or rectum,	GAD scored>9	General Anxiety Disorder-Scale	Moderate	Low	Low	302 (63)
Goncalves 2008 (73)	UK	Longitudinal	61±12	Women	118	ovarian cancer	Anxiety Score>= 8	HADS	Moderate	Low	Moderate	118 (65)**
Grassi 2009 (74)	Italy	Unknown	7.5 ± 11.3	76.1% women	109	Breast, Gastrointestinal, Lung, Genitourinary, Blood and other	Anxiety	ICD-10	Moderate	Low	Low	119 (3)
Hall 1999 (75)	UK	Unknown	<75	Women	266	Breast Cancer	Anxiety Score>= 8	HADS	Low	Low	Low	266 (105)
Hassan 2015 (76)	Malaysia	Cross-sectional	≥20	Women	205	Breast Cancer	Anxiety Score>= 8	HADS	Low	Low	Low	205 (65)
Hegel 2006 (77)	USA	Unknown	57.4±12.3	Women	236	Breast Cancer	GAD Score>= 8 PTSD	Patient Health Questionnaire; Primary Care PTSD Screen	Low	Low	Low	GAD 236 (24) PTSD 236 (24)
Hervouet 2005 (78)	Canada	Cross-sectional	Mean>64	Men	861	Prostate Cancer	Anxiety Score>=7	HADS	Low	Low	Low	861 (204)
Hopwood 2010 (79)	UK	Unknown	20-89	Women	2,181	breast cancer	Anxiety	HADS	Low	Low	Low	2181 (705)**
Isa 2013 (152)	Malaysia	Cross-sectional	Unknown	Men	193	Prostate Cancer	Anxiety	Depression Anxiety Stress Scale	Moderate	Low	Low	193 (28)**
Jimenez−Fonseca 2018 (80)	Spain	Cross-sectional	≥18	59.3% women	600	Colon, Breast, Stomach and other	Anxiety Scores T ≥ 67	Brief Symptom Inventory	Low	Low	Low	600 (139)
Kang 2014 (81)	South Korea	Retrospective cohort	≥20	Women	42,190	breast cancer	Anxiety	ICD-10	Low	Low	Low	42190 (2518)
Karakoyun-Celik 2010 (82)	Turkey	Unknown	31-82	Women	120	breast cancer	Anxiety Score>=60	State-Trait Anxiety Inventory	Moderate	Low	Low	120 (23)
Kazlauskiene 2020 (83)	Lithuania	Unknown	21-80	Women	421	breast cancer	PTSD	Event Scale-Revised	Low	Low	Low	421 (185)
Keller 2004 (84)	Germany	Cross-sectional	≥18	39.7% women	189	Colorectal, Gastric/oesophageal, Pancreatic/liver, Soft tissue and other	Anxiety Score>=11	HADS	Low	Low	Low	189 (36)
Kim 2017 (85)	South Korea	Cross-sectional	>18	Men	161	Prostate cancer	Anxiety Score>=8	HADS	Moderate	Low	Low	161 (14)
Kissane 2004 (86)	USA	Unknown	Mean=46 years	Women	503	breast cancer	GAD Panic disorder PTSD	Monash Interview for Liaison Psychiatry	Low	Low	Low	GAD 503 (8) Panic disorder 503 (4) PTSD 503 (7) Phobia– simple 503 (17) Phobia– social 503 (8) Agoraphobia 503 (5)
Köhler 2014 (87)	Germany	Longitudinal	65.3±6.4	Men	329	Prostate cancer	Anxiety Score>=8	HADS	Moderate	Low	Moderate	329 (106)**
Kuhnt 2016 (43)	Germany	Cross-sectional	18-75	51.5% women	2,141	Breast, Prostate, Colon/rectum, Lung, Female genital organs, Haematological cancers, Stomach/oesophagus, Kidney/urinary tract, Head and neck, Bladder, Pancreas, Malignant melanoma and other	Anxiety	International Diagnostic Interview	Low	Low	Moderate	2141 (340)*
Lichtenthal 2009 (88)	USA	Cross-sectional	58.7±12	44% women	289	Lung, colon, breast, pancreatic, Stomach, Esophageal, Brain, Gallbladder and other	GAD Panic disorder PTSD	Structured Clinical Interview	Low	Low	Low	GAD 273 (6) Panic disorder 272 (8) PTSD 273 (7)
Linden 2012 (89)	USA	Cross-sectional	58.9 ±14.6	55% women	9,394	Bone, Breast, Gastrointestinal, Genitourinary, Gynecological, Head and Neck, Hematological, Lung, Neuroendocrine, Prostate, Skin and other	Anxiety	21-item Psychosocial Screen	Low	Low	Low	Total 9394 (1781) Bone 200 (33) Breast 2250 (477) Gastrointestinal 1230 (208) Genitourinary 281 (50) Gynecological 878 (249) Head and Neck 418 (100) Hematological 167 (38) Lung 602 (155) Neuroendocrine 506 (107) Prostate 1497 (112) Skin 459 (57)
Liu 2017 (91)	China	Cross-sectional	29-79	Women	198	ovarian cancer	Anxiety Score>=8	HADS	Moderate	Low	Low	198 (102)**
Liu 2021 (90)	China	Cross-sectional		Women	389	breast cancer	Anxiety Score>=8	HADS	Low	Low	Low	389 (358)**
Love 2002 (92)	Australia	Unknown	<65	Women	303	breast cancer	Anxiety	Monash Interview for Liaison Psychiatry HADS	Low	Low	Low	Total 303 (32) GAD 303 (5) Panic Disorder 303 (4) PTSD 303 (5)
Lueboonthavatchai 2007 (93)	Thailand	Unknown	20-80	Women	300	Breast Cancer	Anxiety Score>=8	HADS	Low	Low	Low	300 (105)**
Mallet 2018 (94)	France	Cross-sectional	≥18	53.5% women	1,300	liver cancer, breast cancer, mouth, tongue, throat or oesophagus, and other	Anxiety	Disabilities Interview Schedule	Low	Low	Low	Any anxiety 1300 (179) Panic disorder 1300 (38) Social anxiety disorder 1300 (41) Specific phobia 1300 (74) Generalised anxiety disorder 1300 (72) PTSD 1300 (93)
Marco 2019 (95)	Australia	Cross-sectional	18-79	52.1% women	1,183	Breast, Prostate, Colorectal, Melanoma, Lung, Gynaecological, Haematological, Head and neck, Urological, Upper GI and other	Anxiety Score>=8	HADS	Low	Low	Low	1183 (248)
Mehnert 2010 (96)	Germany	Unknown	38-83	Men	511	prostate cancer	Anxiety Score>=8 PTSD	HADS Posttraumatic Stress Disorder Checklist	Low	Low	Low	Anxiety** 511 (75) PTSD 511 (22)
van Montfort 2020 (126)	Netherlands	Cross-sectional	68.3±8.6	51% women	130	lung cancer	Anxiety Score>=8	HADS	Moderate	Low	Low	130 (34)
Naser 2021 (98)	Jordan	Cross-sectional	≥18	44.6% women	1,011	blood cancer, colorectal cancer, lung cancer, and other	Anxiety Score>=8 GAD	HADS, Generalized Anxiety Disorder 7-item (GAD-7)	Low	Low	Low	HADS 1011 (193) GAD 1011 (201)
Ng 2013 (100)	Netherlands	Retrospective cohort	70.77 ±12.76	46.8% women	111	terminal cancer	Anxiety	International Classification for Primary Care	Moderate	Low	Low	111 (2)
Ng 2017 (101)	Malaysia	Cross-sectional	≥18	81.5% women	200	Breast, Genitourinary, Gastrointestinal, Hematological, Hepatobiliary–pancreatic and other	Anxiety Score>=7	HADS	Low	Low	Low	200 (72)
Nikbakhsh 2014 (102)	Iran	Cross-sectional	22-88	52% women	150	breast, colorectal, stomach, esophagus, lung, thyroid	Anxiety Score>=8	HADS	Moderate	Low	Low	150 (69)**
Osborne 2003 (103)	Australia	Cross-sectional	23-60	Women	731	breast cancer	Anxiety Score>=8	HADS	Low	Low	Low	731 (328)**
Palmer 2012 (104)	USA	Unknown	18-85	Women	437	breast cancer	generalized anxiety disorder	Structured Clinical Interview	Low	Low	Low	437 (14)
Perry 2018 (105)	USA	Cross-sectional	≥18	Men	212	Prostate Cancer	Anxiety Score>=5	Depression Anxiety Stress Scale	Low	Low	Low	212 (32)
Price 2010 (106)	Australia	Prospective cohort	18-79	Women	798	ovarian cancer	Anxiety Score>=8	HADS	Low	Low	Low	798 (249)**
Prieto 2002 (107)	Spain	prospective	16-65	41.4% women	220	Hematologic cancer	Phobic disorder Generalized anxiety disorder Panic disorder	Interview	Low	Low	Low	Phobic disorder 220 (18) Generalized anxiety disorder 220 (4) Panic disorder 220 (4)
Priscilla 2011 (108)	Malaysia	Cross-sectional	15-78	52% women	105	Hematological cancer	Anxiety	Mini-International Neuropsychiatric Interview	Low	Low	Low	105 (32)
Puigpinós-Riera 2018 (109)	Spain	prospective retrospective	≥18	Women	2,235	breast cancer	Anxiety Score>=8	HADS	Low	Low	Low	2235 (1086)**
Punnen 2013 (110)	USA	prospective cohort		Men	612	Prostate cancer	General Anxiety Disorder	General Anxiety Disorder scale 7	Low	Low	Low	612 (23)**
Rasic 2008 (111)	Canada	Cross-sectional	≥15	56.5% women	863	Unknown	Anxiety	Composite International Diagnostic Interview	Low	Low	Low	Panic attacks 863 (74) Agoraphobia 863 (13) Social phobia 863 (37)
Roth 2006 (112)	USA	Unknown	≥40	Men	367	Prostate cancer	Generalized Anxiety Disorder Clinical anxiety Score>=27	Generalized Anxiety Disorder Questionnaire	Low	Low	Low	Clinical anxiety 367 (39) GAD 367 (46)
Saboonchi 2014 (113)	Sweden	Prospective cohort	51.3±8.1	Women	713	breast cancer	Anxiety Score>=8	HADS	Low	Low	Low	713 (269)**
Saini 2014 (153)	Italy	Unknown	20-80	47.7% women	153	Colon-rectum, Breast, Prostate, Ovary, Bladder, Gastroenteropancreatic Neuroendocrine, Pancreas, Testis, Stomach, Lung, Adrenal cortical, Uterus, Kidney, Head and neck, Thymus, Esophagus, Thyroid	Anxiety Score>=8	HADS	Low	Low	Low	153 (61)**
Sánchez 2020 (115)	Spain	Prospective		Men	184	Prostate Cancer	Anxiety Score>=11	HADS	Low	Low	Low	184 (26)*
Schellekens 2016 (116)	Netherlands	Unknown	64.1 ±8.7	38.2% women	144	lung cancer	Anxiety disorder Specific phobia Generalized anxiety Disorder Panic disorder	Structural Clinical Interview	Low	Low	Low	Any Anxiety disorder 144 (3) Specific phobia 144 (1) Generalized anxiety Disorder 144 (1) Panic disorder 144 (1)
Singer 2008 (117)	Germany	Cross-sectional	≥30	9% women	308	Ambulatory laryngeal cancer	Social phobia PTSD Generalised anxiety disorder	Structured Clinical Interview	Low	Low	Low	Social phobia 308 (1) PTSD 308 (1) GAD 308 (6)
Smith 2006 (118)	UK	Unknown	21-81	50.4% women	381	Breast, Ovarian, Colorectal, Renal, Lymphoma, Malignant melanoma and other	Anxiety Score>=11	HADS	Low	Low	Low	381 (60)
So 2010 (119)	China	cross-sectional	≥18	Women	218	breast cancer	Anxiety Score>=7	HADS	Low	Low	Low	218 (46)
Spencer 2010 (120)	USA	Longitudinal	≥20	49.8% women	635	Lung, Colon, Pancreatic, Breast Cancer and other	Anxiety PTSD Panic Disorder Generalized Anxiety Disorder	Structured Clinical Interview	Low	Low	Low	Anxiety 635 (48) Panic Disorder 635 (19) PTSD 635 (20) GAD 635 (19)
Stark 2002 (121)	UK	Cross-sectional	22-81	39.9% women	178	Unknown	Anxiety Score>=7	HADS	Low	Low	Low	178 (85)*
Storey 2012 (122)	UK	Cross-sectional	54-95	Men	160	Prostate cancer	Anxiety Score>=9	HADS	Low	Low	Low	160 (30)
Tan 2014 (123)	China	Cross-sectional	53.8±15.2	Women	180	breast cancer	Anxiety Score>=8	HADS	Low	Low	Low	180 (38)
Tavoli 2007 (124)	Iran	Cross-sectional	19-76	44% women	142	gastrointestinal cancer	Anxiety Score>=8	HADS	Moderate	Low	Low	142 (67)
Unseld 2019 (125)	Austria	cross-sectional	18-88	49.6% women	1,017	Brain, Pancreas, Hematological, Female genital organs, Lung, Stomach/esophagus, Head and neck, Soft tissue, Breast, Testis, Kidney/urinary tract/bladder, Colon/rectum, Hepatobiliary, Malignant melanoma, Prostate and other	Anxiety Score>=8 PTSD	HADS	Low	Low	Low	Total 1017 (375)** Men 511 (147) Women 504 (226) PTSD Total 1017 (322) Men 511 (125) Women 504 (196)
Vehling 2017 (127)	Germany	Cross-sectional	56.7 ±11.6	50.5% women	430	Breast, Prostate, Hematological, Gastrointestinal, Gynecological, Lung, and other	Anxiety	Composite International Diagnostic Interview	Low	Low	Low	430 (49)
Vin-Raviv 2013 (129)	USA	Unknown		Women	1,139	Breast cancer	PTSD	Impact of Event Scale	Low	Low	Low	1139 (262)
Vin-Raviv 2015 (128)	USA	cross-sectional	40-85	Women	4,164	breast cancer	Anxiety	ICD-9	Low	Low	Low	4164 (172)
Vodermaier 2011 (130)	Canada	Unknown		65% women	3,850	Lung, breast, prostate, gastrointestinal	Anxiety	21-item Psychosocial Screen for Cancer	Low	Low	Low	Total 3850 (716) Men 1337 (155) Women 2513 (563) Gastrointestinal cancer 839 (140) Lung cancer 374 (99) Breast cancer 1997 (427) Prostate cancer 640 (51)
Watts 2015 (131)	UK	cross-sectional	51-86	Men	313	Prostate cancer	Anxiety Score>=8	HADS	Low	Low	Low	313 (73)
Wen 2017 (132)	China	Survey		Women	123	Ovarian Cancer	Anxiety Score>=11	HADS	Moderate	Low	Low	123 (44)
Wiechno 2013 (133)	Poland	Unknown	50-80	Men	149	prostate cancer	Anxiety Score>=8	HADS	Low	Low	Low	149 (53)**
Wilson 2007 (134)	Canada	Unknown	26-93	55.6% women	381	Unknown	Anxiety	Primary Care Evaluation of Mental Disorders	Low	Low	Low	Any anxiety disorder 381 (53) Panic disorder 381 (21) Generalized anxiety Disorder 381 (22) Anxiety disorder not otherwise specified 381 (18) Anxiety disorder secondary to a general medical condition 381 (7)
Yang 2014 (137)	China	Cross-sectional	49.16 ±10.11	Women	224	Cervical Cancer	Anxiety Score>=8	HADS	Low	Low	Moderate	224 (147)**
Yang 2016 (136)	China	Cross-sectional	18-79	29.4% women	489	Bladder and Kidney Cancer	Anxiety Score>=50	Zung Self-Rating Anxiety Scale PTSD Checklist-Civilian Version	Low	Low	Moderate	Anxiety 489 (339) PTSD 489 (123)
Yang 2017 (135)	Sweden	Cohort	20-80	Women	53,191	Breast cancer	Anxiety	ICD-10	Low	Low	Low	53191 (975)
Yektatalab 2020 (138)	Iran	Cross-sectional	28-76	Women	261	Breast cancer	Anxiety	State−Trait Anxiety Inventory	Low	Low	Low	261 (181)
Zhang 2010 (139)	USA	Cohort	≥65	53.4% women	56,182	Colorectal Cancer	Anxiety	ICD-9	Low	Low	Low	56182 (580)

*One time point selected; **tow cut off point HADS= HADS.

### Quality of studies

Examination of the results in selective bias showed that except for 2 studies that had a high bias, the rest of the studies had a low and moderate bias. All studies had a low bias in the data collection method. Except for 1 study that had a high bias, the rest of the studies had a low and moderate bias in withdrawals/dropouts and missing.

### Prevalence of anxiety

Prevalence of anxiety symptoms/disorders in cancer patients showed that this prevalence is 23% in the confidence interval between 22-25% (*I* ^2^ = 99.59%). This finding shows that approximately one in four cancer patients was anxious.

**Figure 2:**
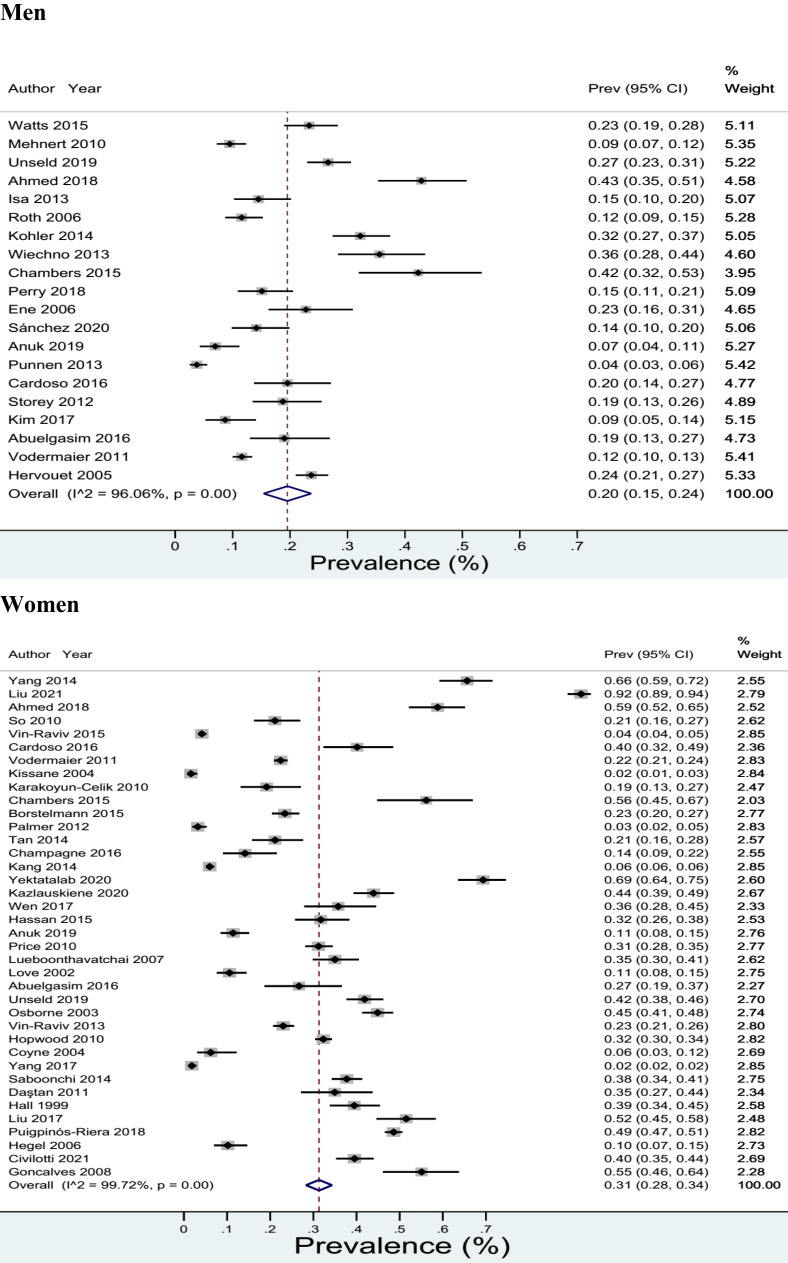
Prevalence of pooled anxiety symptoms/disorders in cancer patients based on sex.

Prevalence of anxiety symptoms/disorders in cancer patients showed that this prevalence was 20% in the confidence interval between 15-24% (*I* ^2^ = 96.06%) in men. Prevalence of anxiety symptoms/disorders in cancer patients showed that this prevalence was 31% in the confidence interval between 28-34% (*I* ^2^ = 99.72%) in women (**Figure 2**).

Prevalence of generalized anxiety disorder in cancer patients showed that this prevalence was 7% in the confidence interval between 5-8% (*I* ^2^ = 95.27%). Prevalence of panic disorder in cancer patients showed that this prevalence was 3% in the confidence interval between 2-4% (*I* ^2^ = 90.43%). Prevalence of PTSD in cancer patients showed that this prevalence was 12% in the confidence interval between 8-16% (*I* ^2^ = 98.91%). Prevalence of specific phobia in cancer patients showed that this prevalence was 4% in the confidence interval between 0-7% (*I* ^2^ = 0%). The prevalence of social phobia in cancer patients showed that this prevalence was 2% in the confidence interval between 0-4% (*I* ^2^ = 90.92%). Prevalence of OCD in cancer patients showed that this prevalence was 0% in the confidence interval between 0-1% (*I* ^2^ = 0%). The prevalence of agoraphobia in cancer patients showed that this prevalence was 2% in the confidence interval between 0-3% (*I* ^2^ = 0%) (**Table 2**).

**Table 2:** Prevalence of different anxiety symptoms/disorders in cancer patients.

Anxiety disorder	Number of Studies	Pooled prevalence	Lower limit	Upper limit	I^2^
GAD	19	0.7	0.5	0.8	95.27%
Panic disorder	11	0.3	0.2	0.4	90.43%
PTSD	14	0.12	0.08	0.16	98.91%
Specific phobia	3	0.4	0	0.7	0%
Social phobia	4	0.2	0	0.4	90.92%
OCD	2	0	0	0.1	0%
Agoraphobia	3	0.2	0	0.3	0%

Highest prevalence of anxiety symptoms/disorders was in ovarian cancer patients showed that this prevalence was 43% in the confidence interval between 31-56% (*I* ^2^ = 93.30%). Lowest prevalence of anxiety symptoms/disorders was in colorectal cancer patients showed that this prevalence was 1% in the confidence interval between 1-1% (*I* ^2^ = 0%) (**Table 3**).

**Table 3:** Prevalence of anxiety symptoms/disorders in cancer patients based on cancer site.

Cancer Site	Number of Studies	Pooled prevalence	Lower limit	Upper limit	I^2^
Hematological cancer	4	0.20	0.06	0.33	96.05%
Colorectal cancer	2	0.01	0.01	0.01	0%
Breast cancer	28	0.27	0.24	0.30	99.77%
Lung cancer	5	0.26	0.10	0.41	98.45%
Prostate cancer	15	0.16	0.12	0.20	96.02%
Ovarian cancer	4	0.43	0.31	0.56	93.30%
Gastrointestinal cancer	3	0.25	0.16	0.35	0%

**Figure 3:**
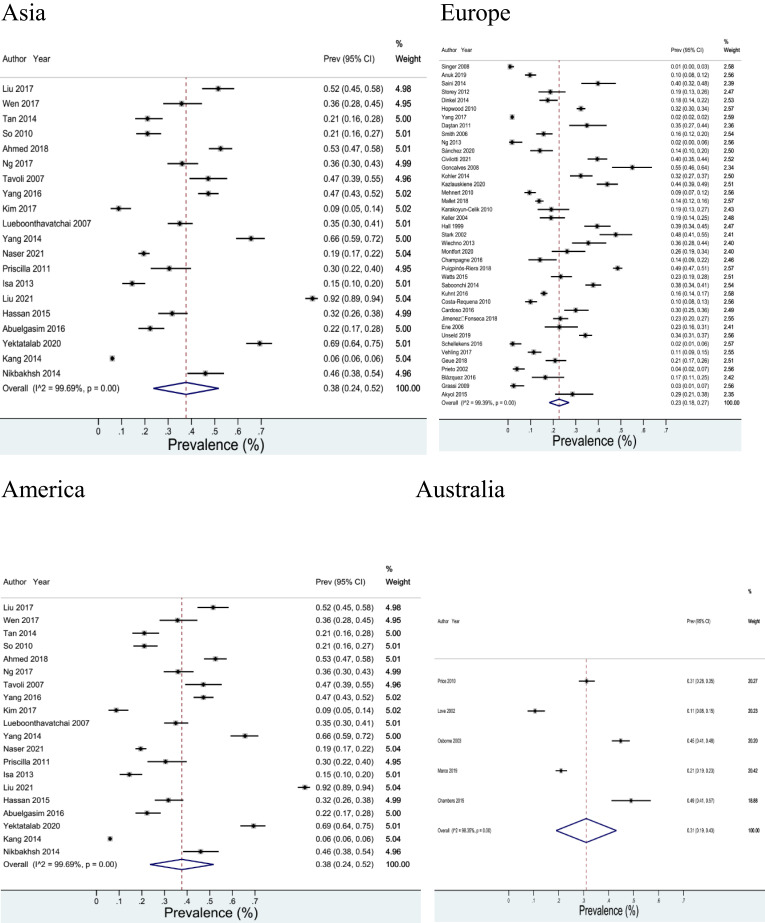
Prevalence of anxiety symptoms/disorders in cancer patients based on continents.

Highest prevalence of anxiety symptoms/disorders in cancer patients was 38% in the confidence interval between 24-52% (*I* ^2^ = 99.69%) in Asia. Lowest prevalence of anxiety in cancer patients was 12% in the confidence interval between 8-15% (*I* ^2^ = 99.55%) in America (**Figure 3**).

### Heterogeneity

To check the heterogeneity of the studies, the *I* ^2^ was used, which showed that it was equal to 99.59 and was high (54). Also, this index was studied at the level of subgroups, but no significant difference was found. χ^2^ as the second test to check heterogeneity was also equal to 20230.12 (d.f 83; p <0.001).

## Discussion

The aim of this study was to investigate the global prevalence of anxiety symptoms/disorders in cancer patients who had cancer at the time of the study. The prevalence of anxiety symptoms/disorders in cancer patients showed that 23% of patients had anxiety, in other words, it can be said that about one in four cancer patients had anxiety symptoms/disorders. This finding indicates a high prevalence of anxiety symptoms/disorders in cancer patients. However, studies in the general population show that the prevalence of anxiety is lower (140). Studies in other countries have also shown that the prevalence of anxiety in the general population is lower (10, 141). Therefore, the findings of the present study highlight the fact that patients with cancer have a higher prevalence of anxiety symptoms/disorders, just as similar studies in cancer patients show a high prevalence of anxiety symptoms/disorders in this population (142, 143). Experiencing anxiety symptoms/disorders after being diagnosed with cancer can be a common process and reaction that a person experiences (144). Cancer, on the other hand, can be considered a traumatic event, and as stated in the etiology of anxiety symptoms/disorders, stressful events can lead to anxiety (145).

Another finding from the current study was that the prevalence of anxiety symptoms/disorders in men with cancer was 20%, while the prevalence was 31% for women. This finding clearly shows that the prevalence of anxiety symptoms/disorders in women with cancer is almost one-third higher than men with cancer. In this regard, studies have shown that in the general population, the prevalence of anxiety in women is higher than men, accordingly (146), the lifetime prevalence of anxiety was 30.5% for women and 19.2% for men (147). The same finding has been shown in various types of anxiety disorders (panic disorder, agoraphobia, specific phobia, social anxiety disorder, GAD, PTSD, OCD), ie higher prevalence of various types of anxiety disorders in women than men (147–149). Another finding from the current study showed that the highest prevalence of anxiety symptoms/disorders among cancers is in ovarian cancer (43%), followed by breast cancer (27%) and lung cancer (26%). Previous studies have shown that the prevalence of anxiety symptoms/disorders varies according to the cancer site (48, 143, 150). The most common type of anxiety disorder in cancer patients was post-traumatic stress disorder (12%), followed by generalized anxiety disorder (7%).

### Limitations

One strength of this study was that it provided a comprehensive meta-analysis of the prevalence of anxiety based on different types of cancer sites. The second strength was that analyzes were based on a variety of anxiety symptoms/disorders and sex. One methodological limitation was the heterogeneity between the studies included in the meta-analysis, and this could be due to different sources, especially since different tools were used to measure anxiety. Some types of cancer sites could not be studied because the number of studies was small or the site of cancer was not identified. The study of prevalence points (4-weeks, 6-months, 12-months, and lifetime) was not possible in the present study and future studies could be performed in this area. Because the studies did not present these distinctions in the results.

### Clinical implications

Considering the role that physical diseases play in mental symptoms and disorders, it is necessary to pay more attention to the appropriateness of psychological interventions for different groups of physical patients in the protocols related to the promotion of mental health as well as therapeutic interventions. After facing a physical disease, one person can suffer from a range of symptoms or mental disorders. Therefore, it is necessary to pay more attention to treatment based on each person. Therefore, it is necessary to develop diagnostic, therapeutic and educational protocols based on cancer and symptoms/anxiety disorders.

## Conclusion

The present study showed that the prevalence of anxiety symptoms/disorders in cancer patients is very high and this issue can impair their level of health and also affect the effectiveness of therapeutic interventions. Therefore, in addition to conventional interventions for cancer treatment, it is necessary to make psychological interventions to improve the mental health of cancer patients. It is also necessary to note that each person has unique characteristics that can affect health, healthy and unhealthy behaviors. Therefore, in interventions related to prevention and treatment, it is always necessary to pay special attention to the issue of personal characteristics.

## Data Availability

The original contributions presented in the study are included in the article/**Supplementary Material**. Further inquiries can be directed to the corresponding author.
