# Supplementary material for: The prevalence of anxiety symptoms/disorders in cancer patients: a meta-analysis
Source: Front Psychiatry. 2024 Nov 15;15:1422540. doi: 10.3389/fpsyt.2024.1422540 (PMC11605443; doi:10.3389/fpsyt.2024.1422540)
Supplement: Supplementary file 1 [file DataSheet1.docx]

**Appendix 1:** Keywords used for PubMed and Scopus search until September 2021.

| Search | Query |
| --- | --- |
| PubMed | 123,092 |
| #1 | Cancer [Mesh] OR Cancer [Text Word] OR Bladder cancer [Mesh] OR Bladder cancer [Text Word] OR Lung cancer [Mesh] OR Lung cancer [Text Word] OR Brain cancer [Mesh] OR Brain cancer [Text Word] OR Melanoma [Mesh] OR Melanoma [Text Word] OR Breast cancer [Mesh] OR Breast cancer [Text Word] OR Non-Hodgkin lymphoma [Mesh] OR Non-Hodgkin lymphoma [Text Word] OR Cervical cancer [Mesh] OR Cervical cancer [Text Word] OR Ovarian cancer [Mesh] OR Ovarian cancer [Text Word] OR Ovarian cancer [Text Word] OR Undergoing chemotherapy [Text Word] |
| #2 | Agoraphobia [Mesh] OR Agoraphobia [Text Word] OR Neurotic Disorders [Mesh] OR Neurotic Disorders [Text Word] OR Obsessive-Compulsive Disorder [Mesh] OR Obsessive-Compulsive Disorder [Text Word] OR Hoarding Disorder [Mesh] OR Hoarding Disorder [Text Word] OR Phobic Disorders [Mesh] OR Phobic Disorders [Text Word] OR Social Phobia [Mesh] OR Social Phobia [Text Word] OR generalized anxiety disorder [Mesh] OR generalized anxiety disorder [Text Word] OR post-traumatic stress disorder [Mesh] OR post-traumatic stress disorder [Text Word] OR phobia [Mesh] OR phobia [Text Word] OR specific phobia [Mesh] OR specific phobia [Text Word] OR Panic Disorder [Mesh] OR Panic Disorder [Text Word] OR Obsessive-Compulsive [Mesh] OR Obsessive-Compulsive [Text Word] OR Neurosis [Mesh] OR Neurosis [Text Word] OR Obsessive-Compulsive Neurosis [Mesh] OR Obsessive-Compulsive Neurosis [Text Word] OR GAD [Mesh] OR GAD [Text Word] OR PTSD [Mesh] OR PTSD [Text Word] OR fear [Mesh] OR fear [Text Word] OR Panic [Mesh] OR panic [Text Word] OR anxiety [Mesh] OR anxiety [Text Word] OR Post-Traumatic [Mesh] OR Post Traumatic [Text Word] OR mental disorders [Mesh] OR mental disorders [Text Word] OR Stress [Mesh] OR Stress [Text Word] OR psychiatric disorders [Mesh] OR psychiatric disorders [Text Word] OR Mental illness [Mesh] OR Mental illness [Text Word] |
| Final | #1 AND #2 |
| Scopus | 136,077 |
| #1 | "Cancer" OR "Bladder cancer" OR "Lung cancer" OR "Brain cancer" OR "Melanoma" OR "Breast cancer" OR "Non-Hodgkin lymphoma" OR "Cervical cancer" OR "Ovarian cancer" OR "Ovarian cancer" OR "Undergoing chemotherapy" |
| #2 | "Agoraphobia" OR "Anxiety Separation" OR "Neurotic Disorders" OR "Obsessive-Compulsive Disorder" OR "Hoarding Disorder" OR "Phobic Disorders" OR "Social Phobia" OR "generalized anxiety disorder" OR "post-traumatic stress disorder" OR "phobia" OR "specific phobia" OR "Panic Disorder" OR "Obsessive-Compulsive" OR "Neurosis" OR "Obsessive-Compulsive Neurosis" OR "GAD" OR "PTSD" OR "fear" OR "panic" OR "anxiety" OR "Post-Traumatic" OR "mental disorders" OR "Stress" OR "psychiatric disorders" OR "Mental illness" OR "Mental Disorders" OR "Psychiatric disorders" |
| Final | #1 AND #2 |
