# Supplementary material for: The prevalence of anxiety symptoms/disorders in cancer patients: a meta-analysis
Source: Front Psychiatry. 2024 Nov 15;15:1422540. doi: 10.3389/fpsyt.2024.1422540 (PMC11605443; doi:10.3389/fpsyt.2024.1422540)
Supplement: Supplementary file 2 [file DataSheet2.docx]

**Appendix 2:** Quality assessment

| **Type of bias** | **Criteria definition** | **Classification (potential for bias)** |
| --- | --- | --- |
|  |  |  |
| Selection bias | Sampling method of the study population, representativeness (response rate, difference between responders and non-responders, investigate and control of variables in case of difference between responders and non-responders) | **Low:** Target population defined as representative of the general population or subgroup of the general population (specific age group, women, men, specific geographic area, and specific occupational group) and response rate is 80% or more.  **Moderate:** Target population defined as somewhat representative of the general population, a restricted subgroup of the general population, response rate 60%-79%.  **High:** Target population defined as “self-referred”/ volunteers, response rate less than 60%. |
| Data collection method | Clear definition of outcome Standard method for outcome assessment  Valid and reliable assessment of outcome | **Low:** Valid and reliable tools for data collection.  **Moderate:** Valid and not reliable tools or reliability is not described.  **High:** Without validity and reliability or both reliability and validity are not described. |
| Withdrawals and drop-outs | Withdrawals and drop-out rates  Size of missing data | **Low:** Follow up participation rate of 80% or higher or missing data on less than 20%.  **Moderate:** Follow up participation rate of 60%-79%, or missing data on 20%-40%.  **High:** Follow up participation rate of less than 60%, or missing data on more than 40%. |
